# *Legionella pneumophila* couples fatty acid flux to microbial differentiation and virulence

**DOI:** 10.1111/j.1365-2958.2008.06593.x

**Published:** 2009-03

**Authors:** Rachel L. Edwards, Zachary D. Dalebroux, Michele S. Swanson

**Affiliations:** 1 Cellular and Molecular Biology Program and University of Michigan Medical School, Ann Arbor, MI, USA; 2 Department of Microbiology and Immunology, University of Michigan Medical School, Ann Arbor, MI, USA

## Abstract

During its life cycle, *Legionella pneumophila* alternates between at least two phenotypes: a resilient, infectious form equipped for transmission and a replicative cell type that grows in amoebae and macrophages. Considering its versatility, we postulated that multiple cues regulate *L. pneumophila* differentiation. Beginning with a Biolog Phenotype MicroArray screen, we demonstrate that excess short-chain fatty acids (SCFAs) trigger replicative cells to cease growth and activate their panel of transmissive traits. To co-ordinate their response to SCFAs, *L. pneumophila* utilizes the LetA/LetS two-component system, but not phosphotransacetylase or acetyl kinase, two enzymes that generate high-energy phosphate intermediates. Instead, the stringent response enzyme SpoT appears to monitor fatty acid biosynthesis to govern transmission trait expression, as an altered distribution of acylated acyl carrier proteins correlated with the SpoT-dependent differentiation of cells treated with either excess SCFAs or the fatty acid biosynthesis inhibitors cerulenin and 5-(tetradecyloxy)-2-furoic acid. We postulate that, by exploiting the stringent response pathway to couple cellular differentiation to its metabolic state, *L. pneumophila* swiftly acclimates to stresses encountered in its host or the environment, thereby enhancing its overall fitness.

## Introduction

*Legionella pneumophila* is a promiscuous, Gram-negative pathogen commonly found in freshwater systems. In these environments, *L. pneumophila* efficiently parasitizes many different species of amoebae and protozoa (Fields et al., 2002). Moreover, *Legionella* can establish biofilms in both natural and potable water systems, which serve as reservoirs of contamination (Fields et al., 2002). If susceptible individuals aspirate bacteria-laden aerosols, the pathogen can colonize the alveolar macrophages to cause the severe pneumonia Legionnaires’ disease. Due to the disparate conditions under which *L. pneumophila* can survive, the bacteria must utilize mechanisms to monitor their milieu and swiftly acclimate to their surroundings.

To tolerate environmental fluctuations, many bacteria alter their cellular physiology and morphology, a process known as differentiation. The sexually transmitted bacterium *Chlamydia trachomatis* alternates between an extracellular, metabolically inert elementary body required for transmission and an intracellular, metabolically active reticulate body that undergoes repeated cycles of cell division (Samuel et al., 2003; Abdelrahman and Belland, 2005). Likewise, the aetiologic agent of human Q fever, *Coxiella burnetii*, differentiates between a replicative large cell variant and a resilient small cell variant (Heinzen et al., 1999). Within biofilm communities, the opportunistic pathogen *Pseudomonas aeruginosa* alternates between distinct motile and non-motile cell types (Purevdorj-Gage et al., 2005). By employing cellular differentiation, bacterial pathogens can evade host defence mechanisms and promote self-preservation.

Ground-breaking work by Rowbotham revealed that, within amoebae, *L. pneumophila* also exhibits two distinct phenotypes: a non-motile, thin-walled replicative form and a motile, thick-walled infectious form that contains stores of an energy-rich polymer (Rowbotham, 1986). Corroborating these early findings, subsequent genetic and molecular studies determined that the replicative and transmissive phases of the *L. pneumophila* life cycle are reciprocal (Byrne and Swanson, 1998; Hammer and Swanson, 1999; Alli et al., 2000; Watarai et al., 2001; Wieland et al., 2002; Molofsky and Swanson, 2003). The current model suggests that when phagocytic cells engulf transmissive *L. pneumophila*, the bacteria avoid lysosomal degradation by establishing vacuoles isolated from the endosomal network, a process mediated by the Dot/Icm type IV secretion system and its substrates, as well as by vesicles shed from the outer membrane (Berger and Isberg, 1993; Berger et al., 1994; Joshi et al., 2001; Segal et al., 2005; Fernandez-Moreira et al., 2006). If conditions in the vacuole are favourable, the RNA-binding protein CsrA and the sRNA chaperone Hfq repress transmissive traits, enabling *L. pneumophila* to replicate profusely (Fettes et al., 2001; Molofsky and Swanson, 2003; McNealy et al., 2005). Once nutrient supplies are exhausted, replication halts, and the progeny initiate a global change in their physiology known as the stringent response (Hammer and Swanson, 1999; Zusman et al., 2002). This pathway generates the alarmone ppGpp, which co-ordinates bacterial differentiation. In particular, a major shift in the *L. pneumophila* transcriptional profile is mediated by alternative sigma factors, while the LetA/LetS two-component system relieves CsrA repression on transmissive traits (Hammer and Swanson, 1999; Hammer et al., 2002; Molofsky and Swanson, 2003; Bruggemann et al., 2006). As a consequence, *L. pneumophila* expresses a panel of traits that are vital for dissemination, including cytotoxicity, motility and lysosome evasion (Bachman and Swanson, 2001; 2004a,b; Hammer et al., 2002; Lynch et al., 2003; Jacobi et al., 2004). In addition, *L. pneumophila* may further develop into the highly resilient and infectious mature intracellular form under defined conditions (Faulkner and Garduno, 2002; Garduno et al., 2002). Eventually, the exhausted host cell lyses, releasing transmissive *L. pneumophila* into the environment, which can then initiate subsequent rounds of infection.

Amino acid concentrations appear to be a critical metabolic cue, as fluctuations in their availability alter the developmental state of the microbe. For example, intracellular *L. pneumophila* rely on PhtA, a transporter of the Major Facilitator Superfamily, to gauge whether the threonine supply is sufficient to sustain growth (Sauer et al., 2005). Furthermore, macrophages require the amino acid transporter SLC1A5 to support replication of intracellular *L. pneumophila* (Wieland et al., 2005). Studies of broth cultures predict that when amino acid supplies are depleted, uncharged tRNAs accumulate, and the stringent response enzyme RelA produces the ppGpp signalling molecule which triggers *L. pneumophila* differentiation (Hammer and Swanson, 1999; Zusman et al., 2002).

Because *Legionella* persist in diverse environments, we postulated that signals other than amino acids also induce their differentiation. Indeed, for transmission between macrophages, *L. pneumophila* requires SpoT (Dalebroux et al., 2009), a second ppGpp synthetase known to equip *Escherichia coli* to generate the alarmone in response to a variety of stresses, such as phosphate starvation or inhibition of fatty acid biosynthesis (Seyfzadeh et al., 1993; Gong et al., 2002; Zusman et al., 2002; Magnusson et al., 2005; Battesti and Bouveret, 2006). By screening hundreds of metabolites via Biolog Phenotype MicroArrays, and then applying a series of pharmacological, biochemical and genetic tests, we determined that, in response to perturbations in fatty acid biosynthesis, replicative *L. pneumophila* rely on SpoT to activate the stringent response pathway and co-ordinately express transmissive traits, thereby coupling phase differentiation to their metabolic state.

## Results

### *Biolog Phenotype MicroArrays identify novel cues of* L. pneumophila *differentiation*

To identify signals that trigger *L. pneumophila* differentiation, we employed Biolog Phenotype MicroArrays to screen various sources of carbon, nitrogen, sulphur and phosphorous. Exponential (E) *L. pneumophila* carrying a *gfp* reporter for flagellin, a marker of transmissive bacteria (Table 1), were cultured in the plates, and their relative fluorescence was monitored. Of the 387 compounds screened, only 22 (6%) induced *flaAgfp* expression prematurely (Table 2). Among these were deoxyadenosine, deoxyribose, 2-deoxy-d-glucose 6-phosphate, dihydroxy-acetone, nitrite, hydroxylamine, parabanic acid and methionine–alanine dipeptide. However, the predominant class of compounds (12 of 22) was carboxylic acids. In particular, the four short-chain fatty acids (SCFAs) – formic, acetic, propionic and butyric acid, and the medium chain fatty acid caprioc acid all triggered *flaAgfp* expression. Also eliciting a positive response were two detergents, Tween 20 and Tween 80; however, both detergents also contain the carboxylic acid groups lauric and oleic acid respectively. Indeed, when exposed to 5 mM lauric acid, a 12 carbon carboxylic acid, *L. pneumophila* also stopped replicating and expressed *flaAgfp* (data not shown). However, the response to lauric acid was slower and less robust than to the SCFAs, likely due to the requirement for receptor-mediated transport across the *L. pneumophila* membrane and perhaps the time needed for the fatty acid to be degraded via b-oxidation. Interestingly, high concentrations of SCFAs inhibit the growth of many microorganisms (Bohnhoff et al., 1964), including *L. pneumophila* (Warren and Miller, 1979). Moreover, acetate, propionate and butyrate regulate *Salmonella typhimurium* invasion gene expression *in vitro* at concentrations that correlate with their abundance in the intestinal tract (Lawhon et al., 2002). Therefore, we postulated that *L. pneumophila* monitors SCFAs to co-ordinate its life cycle.

### *Excess short chain fatty acids inhibit* L. pneumophila *growth and induce motility*

**Fig. 1.**
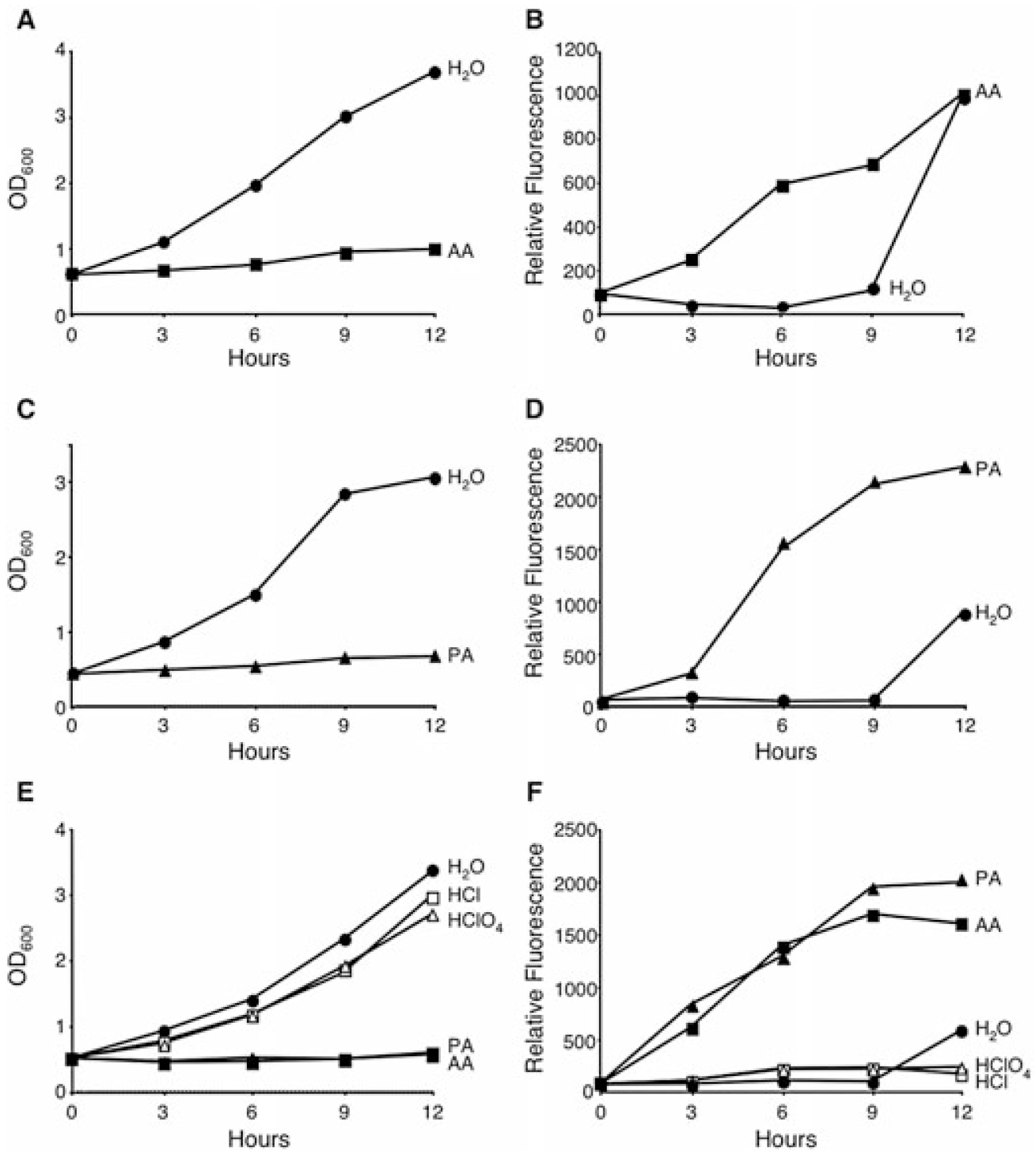
Growth inhibition and the premature expression of motility are specific to fatty acid addition. WT *L. pneumophila* carrying the *flaAgfp* reporter construct were cultured to the E phase and then supplemented with 10 mM acetic acid (AA; closed squares), propionic acid (PA; closed triangles), hydrochloric acid (HCl; open squares) or perchloric acid (HClO_4_; open triangles). At the times indicated, the optical density (A, C, E) and relative fluorescence (B, D, F) of samples were analysed. For all experiments, E cultures supplemented with water (H_2_O; closed circles) served as a negative control. Shown are representative graphs from three or more independent experiments in which the mean fold change in fluorescence at 9 h ± SEM when compared with H_2_O control was: AA = 15.7 ± 3.1; PA = 50.5 ± 15.2; HCl = 0.87 ± 0.48; HClO_4_ = 1.0 ± 1.0.

Acetic and propionic acid, which were identified in the Phenotype MicroArray screen, were selected for further analysis as previous data indicate that both regulate virulence genes in *S. typhimurium*, albeit with opposite effects (Lawhon et al., 2002). As predicted, when E cultures were treated with either 10 mM acetic or propionic acid, *L. pneumophila* immediately stopped replicating (Fig. 1A and C) and activated the *flaA* promoter (Fig. 1B and D). The growth inhibition of *L. pneumophila* treated with the SCFAs was not attributed to a loss in viability, as judged by enumerating colony-forming units (cfu) (data not shown). In contrast, control cultures supplemented with water did not induce the *flaA* promoter or halt replication until the transition to the post-exponential (PE) phase, which occurred at 9 h (corresponds to OD_600_ ≈ 3.4; Fig. 1, circles). Titration experiments analysing a range from 2.5 to 20 mM demonstrated that 10 mM SCFAs was optimal, as lower concentrations failed to significantly activate the *flaA* promoter or inhibit growth (data not shown).

To determine whether the response by *L. pneumophila* to SCFAs was a consequence of alterations in pH, E bacteria were instead treated with two inorganic acids. When added to concentrations of 1.25–20 mM, neither hydrochloric nor perchloric acid triggered growth inhibition or induction of the *flaAgfp* promoter (Fig. 1E and F; data not shown). Moreover, the pH of *L. pneumophila* cultures supplemented with acetic or propionic acid did not differ significantly from those supplemented with water, nor did the pH of the treated cultures vary detectably over the course of the experiment (data not shown). Finally, when E cultures were supplemented with non-acidic forms of acetate, *L. pneumophila* stopped replicating and activated the flagellin promoter. For example, when supplemented with 10 mM calcium acetate for 6 h, E cultures showed a 28-fold induction of *flaAgfp* when compared with water control (data not shown). Therefore, *L. pneumophila* respond to a signal generated by SCFAs that is distinct from pH.

### *Fatty acid supplementation stimulates* L. pneumophila *differentiation*

**Fig. 2.**
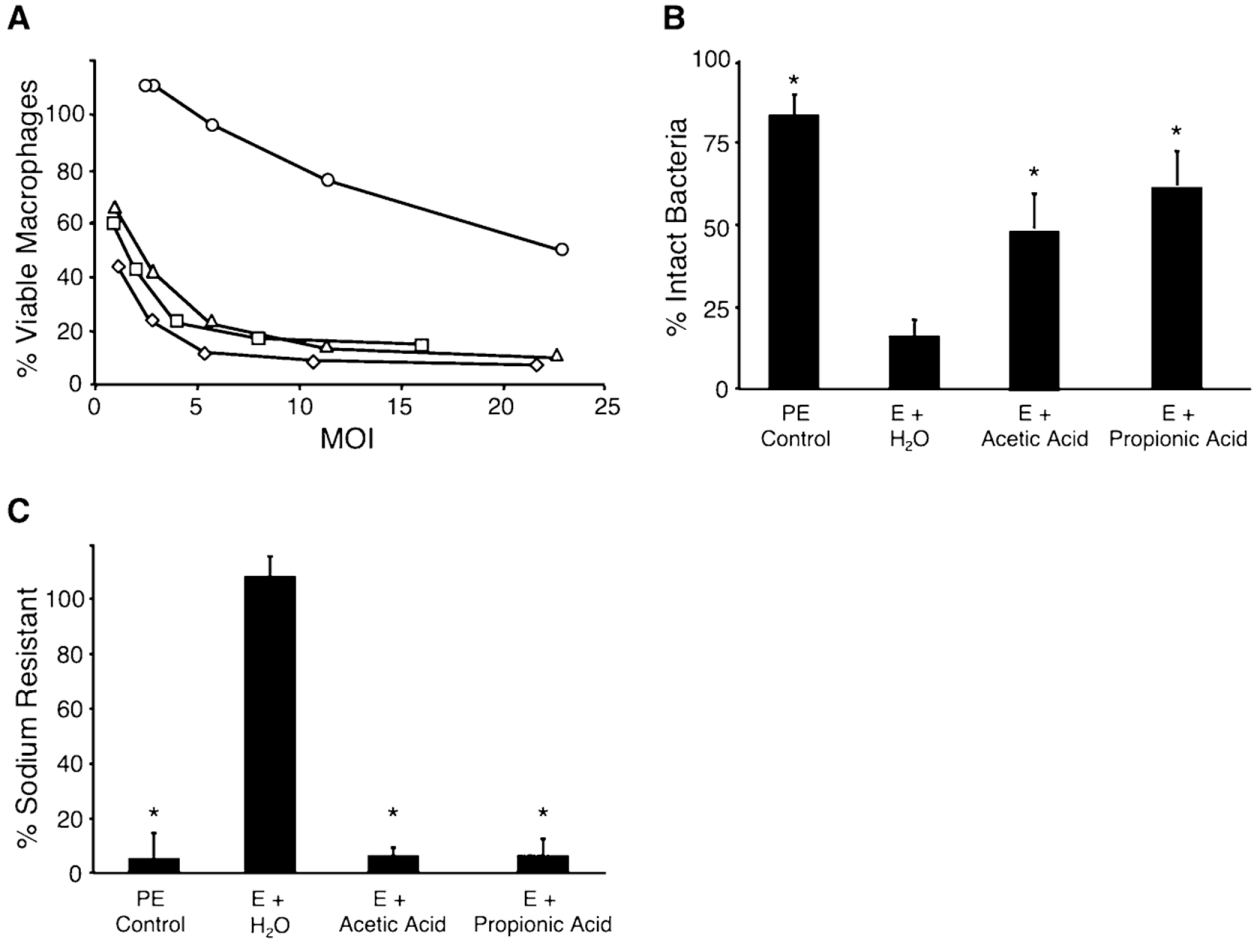
Fatty acid supplementation of WT *L. pneumophila* induces the early expression of multiple transmissive phase phenotypes. A. Macrophage viability was assessed by alamarBlue reduction following a 1 h incubation with PE (diamonds) or E cultures supplemented with water (circles), acetic acid (squares) or propionic acid (triangles). Shown is a representative graph from three independent experiments preformed in triplicate. B. Lysosome evasion following a 2 h incubation was quantified by fluorescence microscopy as the per cent of intact bacteria. Displayed are the means from duplicate samples in three independent experiments. Error bars indicate SD and asterisks designate significant differences (*P*<0.01) when compared with water control. C. The mean percentage of sodium-resistant bacteria ± SD was calculated from duplicate samples in three independent experiments. Asterisks denote statistically significant differences (*P*<0.01) when compared with water control.

We next investigated whether SCFAs trigger other *L. pneumophila* transmissive phase phenotypes, including motility, cytotoxicity to phagocytic cells, avoidance of lysosomal degradation and sodium sensitivity (Byrne and Swanson, 1998). As expected from the *flaAgfp* data (Fig. 1), microscopic examination revealed that 10 mM SCFAs induced motility (Table 3). Also, after supplementation with either acetic or propionic acid, E phase *L. pneumophila* became as cytotoxic to macrophages as PE control cultures (Fig. 2A). Importantly, the addition of SCFAs alone was not cytotoxic (Table 3, *letA* and *letS* mutants; data not shown). Further, although only 15% of E phase control *L. pneumophila* avoided degradation, > 50% of those exposed to acetic or propionic acid remained intact (Fig. 2B). Finally, 10 mM acetic or propionic acid also triggered sodium sensitivity in E phase microbes (Fig. 2C). Thus, our Biolog screen accurately predicted that exposure to 10 mM SCFAs induces the *L. pneumophila* transmissive phenotype. Moreover, our studies support previous data that indicate that the *flaAgfp* reporter is a valid marker of *L. pneumophila* differentiation (Hammer and Swanson, 1999; Sauer et al., 2005; Bruggemann et al., 2006). Accordingly, we next investigated two potential modes of action: excess SCFA may either generate high-energy intermediates that activate two-component phosphorelay systems or instead alter fatty acid metabolism.

### *To respond to fatty acids,* L. pneumophila *requires the LetA/LetS two-component system, but not generation of acetyl-phosphate and propionyl-phosphate*

**Fig. 3.**
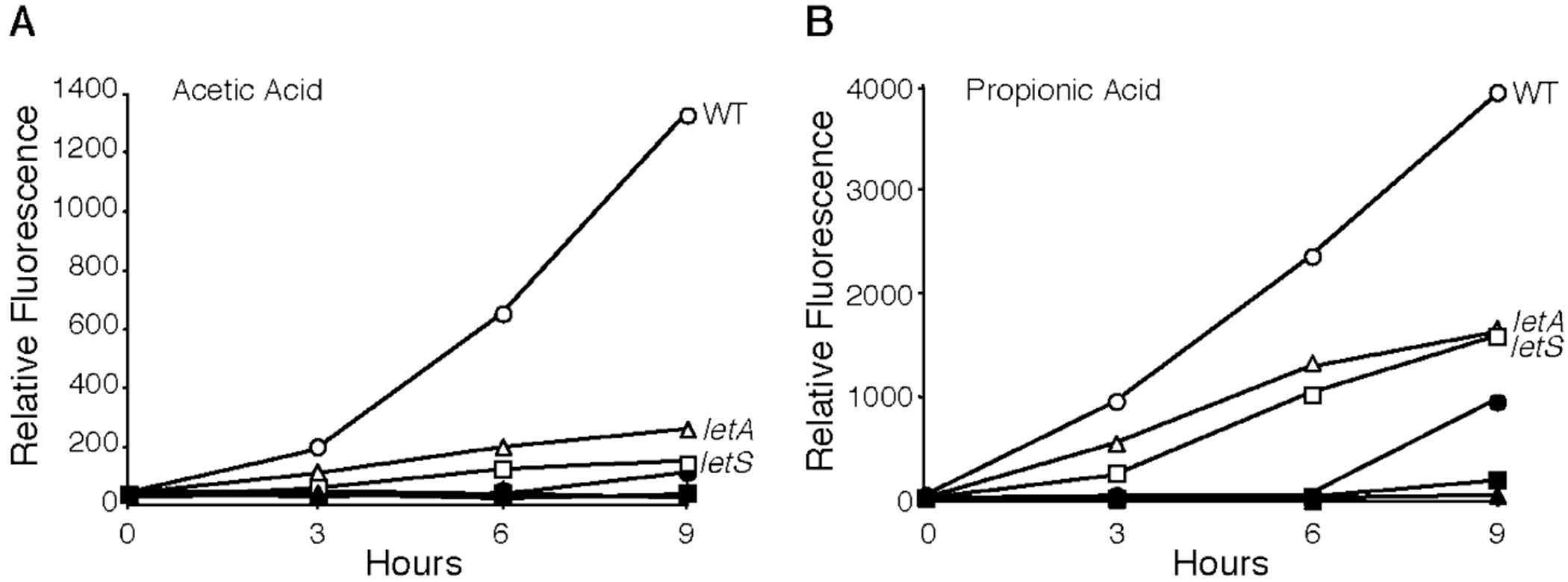
The LetA/LetS signal transduction system is required for full induction of premature motility. E phase WT (circles), *letA* (triangles) or *letS* (squares) *L. pneumophila* containing the *flaAgfp* reporter were supplemented with water (closed symbols), 10 mM acetic acid (open symbols; A) or 10 mM propionic acid (open symbols; B), and their fluorescence analysed at the times indicated. Shown are representative graphs from three independent experiments in which the mean fold change in fluorescence at 6 h ± SEM when compared with H_2_O control was: WT + acetic acid = 39.7 ± 15.7; *letA* + acetic acid = 6.2 ± 2.5; *letS* + acetic acid = 6.7 ± 2.3; WT + propionic acid = 77.0 ± 8.5; *letA* + propionic acid = 59.0 ± 10.0; *letS* + propionic acid = 52.2 ± 13.6.

The *L. pneumophila* two-component system LetA/LetS regulates all known transmissive phase phenotypes (Hammer et al., 2002 Lynch et al., 2003; Broich et al., 2006; Shi et al., 2006). To discern whether the response of *L. pneumophila* to SCFAs depends on this signal transduction system, we exploited *letA* and *letS* mutants. When confronted with SCFAs, the *letA* and *letS* mutants resembled wild-type (WT) *L. pneumophila* by restricting their growth (Table 3). However, *L. pneumophila* required the LetA/LetS system to induce flagellin expression in response to 10 mM acetic acid (Fig. 3A) and also the stronger inducer propionic acid, albeit to a lesser degree (Fig. 3B). The two-component system was also largely required for expression of four other transmissive traits: motility, cytotoxicity, lysosome avoidance and sodium sensitivity (Table 3). Therefore, when *L. pneumophila* encounters a sudden increase in SCFAs, a pathway that includes LetA/LetS co-ordinates bacterial differentiation.

**Fig. 4.**
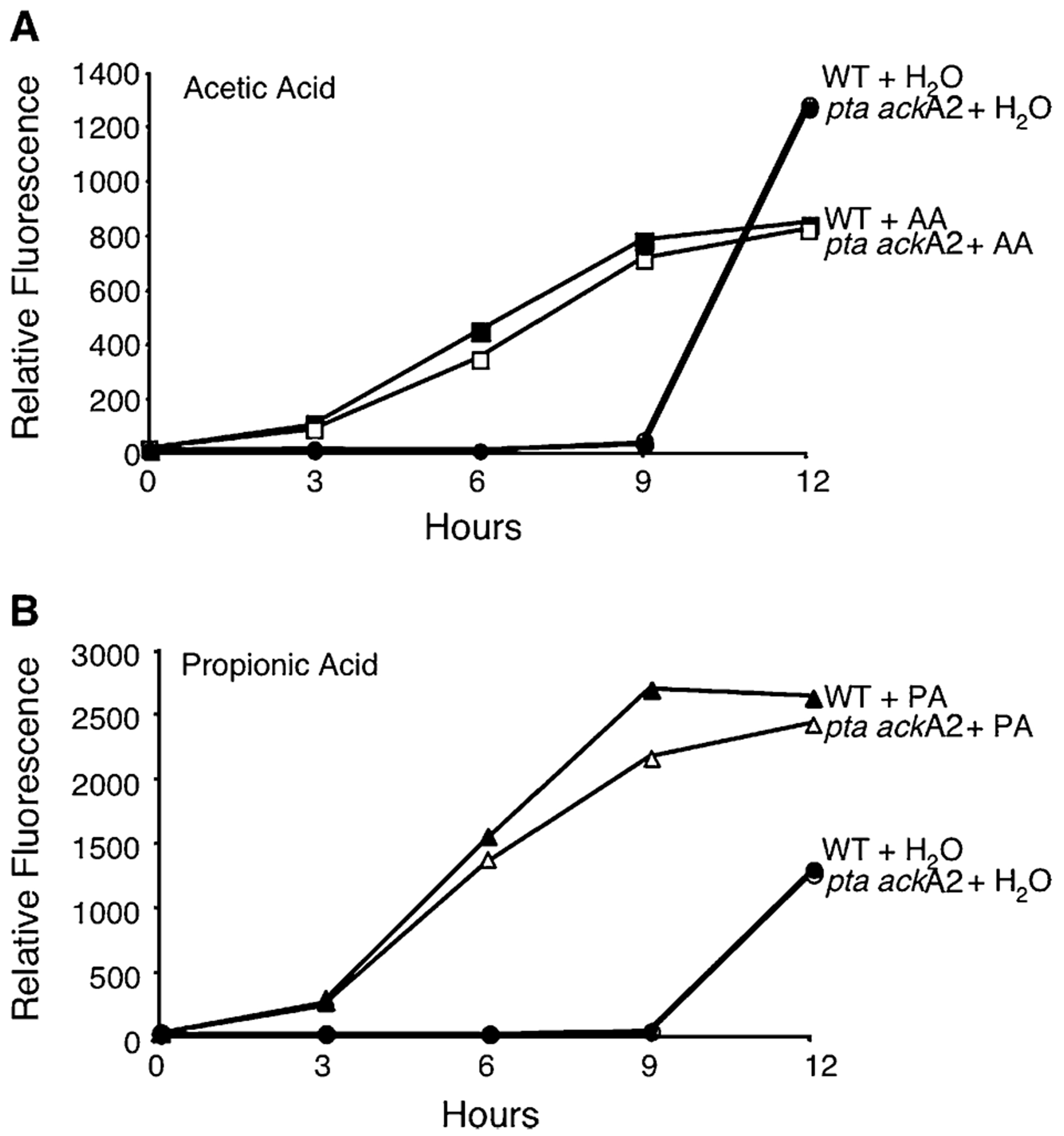
Induction of motility by fatty acid addition is independent of *pta ackA2*. E phase broth cultures of WT (closed symbols) or *pta ackA2* (open symbols) *L. pneumophila* containing pflaG were supplemented with water (H_2_O; circles), 10 mM acetic acid (A; AA: squares), or 10 mM propionic acid (B; PA: triangles), and their relative fluorescence assessed by fluorometry at 3 h intervals. Shown are representative graphs from three experiments in which the mean fold change in fluorescence at 9 h ± SEM when compared with H_2_O control was: WT + AA = 11.9 ± 4.6; *pta ackA2* + AA = 11.9 ± 5.1; WT + PA = 42.0 ± 17.0; *pta ackA2* + PA = 37.5 ± 14.1.

The response regulators of many two-component systems can use the high-energy intermediates acetyl-phosphate and propionyl-phosphate to catalyse their own phosphorylation (Boucher et al., 1994; Lawhon et al., 2002; Wolfe, 2005). Therefore, we tested whether exogenous acetic or propionic acid are first converted to acetyl- and propionyl-phosphate before activating the LetA/LetS signal transduction system by analysing a *L. pneumophila* mutant that lacks the two enzymes that synthesize the phosphate intermediates, phosphotrans-acetylase and acetyl kinase, encoded by the *pta* and *ackA2* genes respectively (McCleary et al., 1993; Wolfe, 2005). By monitoring the activity of the flagellin promoter, it was evident that neither the phosphotransacetylase nor the acetyl kinase enzyme was needed for *L. pneumophila* to differentiate when confronted by excess acetic or propionic acid (Fig. 4). Thus, unlike *Salmonella* and *Bordetella* (Boucher et al., 1994; Lawhon et al., 2002), SCFAs trigger *L. pneumophila* differentiation by a mechanism other than generating acetyl- and propionyl-phosphate intermediates to activate LetA/LetS.

### *Perturbations in fatty acid biosynthesis trigger* L. pneumophila *differentiation*

**Fig. 5.**
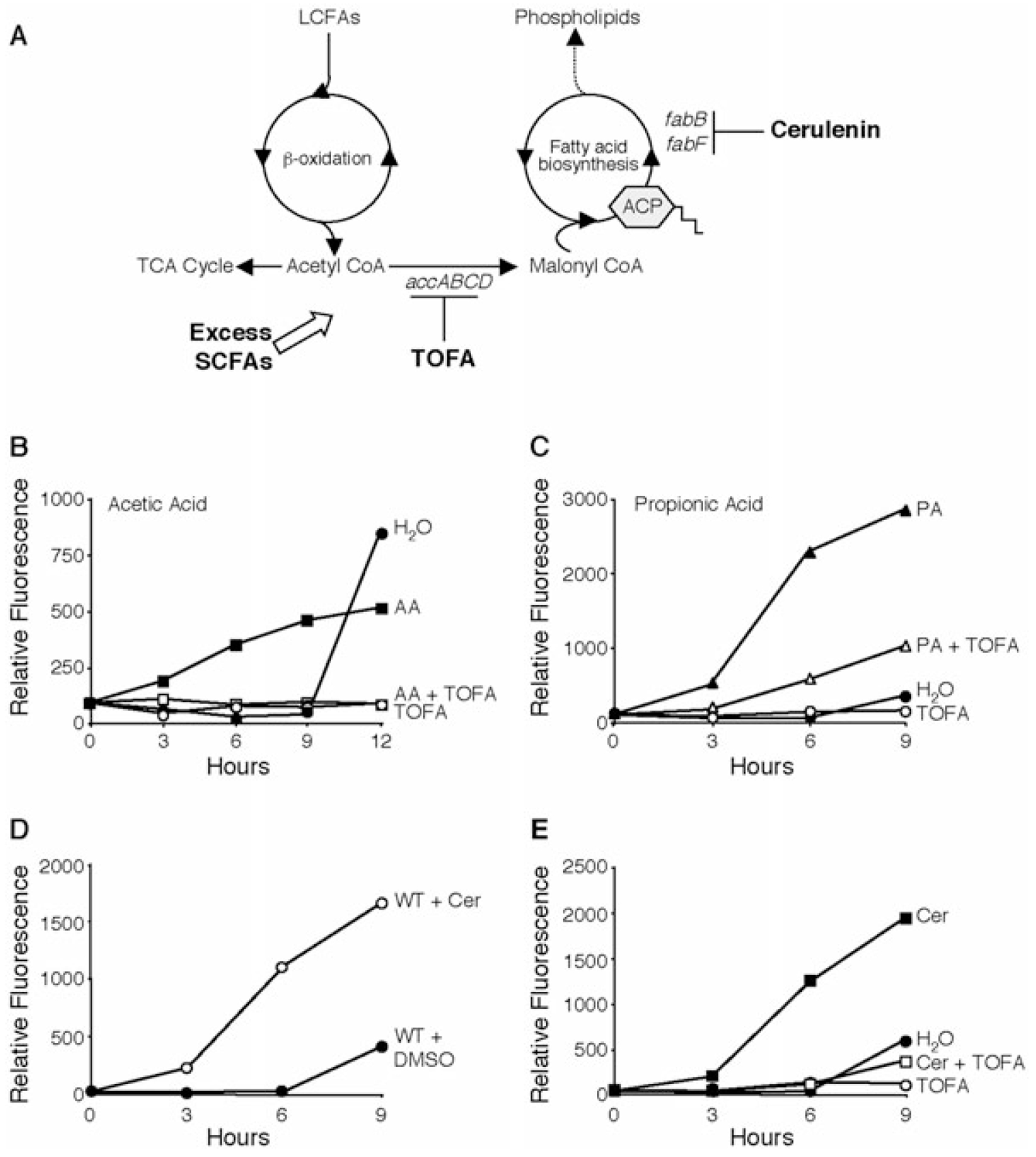
Alterations in fatty acid biosynthesis induce *L. pneumophila* differentiation. A. Schematic of fatty acid metabolism indicating where TOFA and cerulenin inhibitors act. LCFA, long-chain fatty acid. B and C. Inhibition of the conversion of acetyl-CoA to malonyl-CoA abrogates the early differentiation that is triggered by fatty acid supplementation. B. E cultures containing pflaG were supplemented with 10 mM acetic acid (AA; squares) with (open shapes) or without (closed shapes) the acetyl-CoA carboxylase inhibitor TOFA (5 μg ml^−1^; open circles), and the fluorescence was monitored over time. Identical cultures supplemented with water (closed circles) or DMSO (vehicle control, data not shown) were analysed as controls. The graph shown is representative of three independent experiments in which the mean fold change in fluorescence at 9 h ± SEM when compared with H_2_O control was: AA= 10.2 ± 4.1; TOFA = 2.4 ± 1.4; AA+TOFA= 1.6 ± 0.4. C. E cultures containing *flaAgfp* were supplemented with 10 mM propionic acid (PA; triangles) with (open shapes) or without (closed shapes) TOFA (5 μg ml^−1^; open circles) and the fluorescence was monitored over time. Identical cultures supplemented with water (closed circles) or DMSO (vehicle control, data not shown) were analysed as controls. The graph shown is representative of four independent experiments in which the mean fold change in fluorescence at 6 h ± SEM when compared with H_2_O control was: PA = 73.8 ± 16.4; TOFA = 5.3 ± 1.1; PA + TOFA = 21.4 ± 6.2. D. E cultures of WT *L. pneumophila* carrying the *flaAgfp* plasmid were supplemented with the fatty acid biosynthesis inhibitor, cerulenin (Cer, 0.5 μg ml^−1^; open circles) or vehicle control (DMSO, closed circles), and the relative fluorescence monitored over time. The graph shown is representative of four independent experiments in which the mean fold change in fluorescence at 6 h ± SEM when compared with DMSO control was: Cer = 65.0 ± 8.1. E. E phase cultures of WT *L. pneumophila* containing pflaG were supplemented with cerulenin (Cer, 0.5 μg ml^−1^; closed squares) or cerulenin plus TOFA (open squares). Identical cultures treated with water (H_2_O; closed circles) or TOFA alone (open circles) are shown as controls. In three separate experiments, the mean fold change in fluorescence at 6 h ± SEM when compared with H_2_O control was: Cer = 39.8 ± 10.1; TOFA = 3.9 ± 1.1; Cer + TOFA = 6.8 ± 2.0.

To begin to test the hypothesis that SCFA supplements impinge upon either fatty acid degradation or biosynthesis, we tested whether acetic and propionic acid trigger *L. pneumophila* differentiation when the irreversible conversion of acetyl-CoA to malonyl-CoA is blocked. In mammalian cells, the competitive inhibitor 5-(tetradecyloxy)-2-furoic acid (TOFA) blocks the acetyl-CoA carboxylase (ACC) complex (*accA*, *lpg0785*; *accB*, *lpg0463*; *accC*, *lpg0462* and *accD*, *lpg1341*) and prevents acetate from being incorporated into fatty acids (Fig. 5A; Panek et al., 1977; Cook et al., 1978; McCune and Harris, 1979; Magnuson et al., 1993; Pizer et al., 2000; Zhou et al., 2003). Accordingly, malonyl-CoA levels in the cell are significantly reduced and fatty acid biosynthesis is halted. When cultures were simultaneously supplemented with SCFAs and TOFA, the majority of bacteria failed to differentiate (Fig. 5B and C). When treated with TOFA alone, *L. pneumophila* did not differentiate, although their growth was restricted and viability was maintained, as the number of cfu was similar between 0 and 24 h after TOFA treatment (data not shown). Because activity of the ACC complex appeared to be required for SCFAs to initiate *L. pneumophila* differentiation, we deduced that addition of 10 mM acetic or propionic acid likely affects the fatty acid biosynthetic pathway.

As an independent test of this model, we exploited the antibiotic cerulenin, which has been well documented in *E. coli* to irreversibly block two key fatty acid enzymes, FabB (*lpg0102*, *lpg0361* and *lpg0362*) and FabF (*lpg1397*; Fig. 5A; Vance et al., 1972; Omura, 1976; Buttke and Ingram, 1978; Ulrich et al., 1983). Rather than depleting malonyl-CoA, cerulenin causes this precursor to accumulate in the cell (Heath and Rock, 1995). When E phase WT *L. pneumophila* were treated with 0.5 μg ml^−1^ cerulenin, bacterial replication stopped (data not shown) and the *flaA* promoter was activated (Fig. 5D). This response was largely dependent on the LetA/LetS two-component system because WT cultures treated with cerulenin exhibited a 54 ± 15-fold change in fluorescence when compared with the DMSO control sample at 6 h, whereas the signal from *letA* and *letS* cultures increased only 12 ± 2.7-fold and 16 ± 3.9-fold respectively (data reported for each strain are the means ± SEM in three independent experiments). In eukaryotic cells, the simultaneous addition of cerulenin and TOFA decreases malonyl-CoA levels and blocks fatty acid biosynthesis (Pizer et al., 2000). Whereas cerulenin activates the *L. pneumophila flaA* promoter, cultures treated with both cerulenin and TOFA did not differentiate (Fig. 5E), implicating malonyl-CoA accumulation as a prerequisite for the stress response. Taken together, the effects of both SCFA supplementation and the pharmacological inhibitors of particular biosynthetic enzymes indicate that, when fatty acid biosynthesis is disrupted, *L. pneumophila* differentiates to the transmissive phase. Moreover, although it is well documented that in mammalian cells TOFA inhibits the ACC complex (Panek et al., 1977; McCune and Harris, 1979), this is the first indication that TOFA might elicit a similar response in microbes.

### Short chain fatty acid supplements alter the profile of acylated acyl carrier proteins

A critical component of fatty acid and lipid biosynthesis is acyl carrier protein (ACP). In *E. coli*, once ACP is modified by a 4′-phosphopantetheine group, the small, acidic protein carries the growing fatty acid chain through successive rounds of elongation (Magnuson et al., 1993). To ascertain by an independent, biochemical approach whether SCFA supplementation alters the *L. pneumophila* fatty acid biosynthetic pathway, we analysed their acyl-ACP pools.

**Fig. 6.**
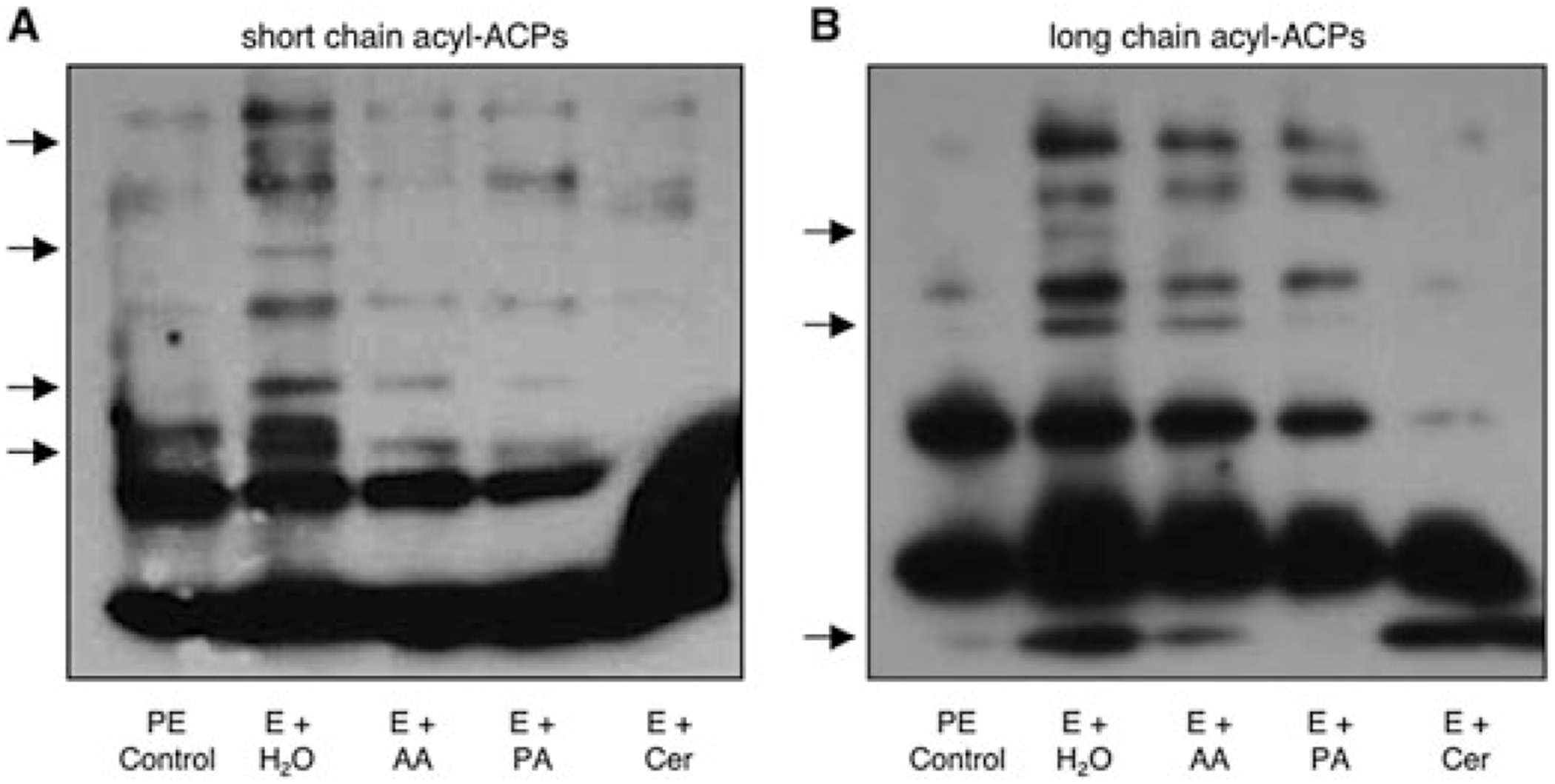
Perturbations in fatty acid biosynthesis alter *L. pneumophila* acyl-ACP profiles. After incubating E phase *L. pneumophila* for 3 h with water (H_2_O), acetic acid (AA), propionic acid (PA), or cerulenin (Cer), acyl-ACPs were purified, separated on 13% short-chain fatty acid (A) or long-chain fatty acid (B) native polyacrylamide gels, and then detected by western analysis. Also shown are acyl-ACP pools from PE bacteria. Arrows denote protein bands that differ between the control and experimental samples. A film representative of three independent experiments is displayed.

When E cultures were supplemented with 10 mM acetic or propionic acid for 3 h, the profiles of acyl-ACPs were significantly different from those supplemented with water (Fig. 6). In particular, cultures treated with the SCFAs resembled the PE control, as similar ACP bands were depleted. A similar pattern was observed after treatment with cerulenin (Fig. 6). Therefore, these biochemical data are consistent with the model that flux in fatty acid biosynthesis triggers *L. pneumophila* differentiation.

### Alterations in the fatty acid biosynthetic pathway stimulates the stringent response

**Fig. 7.**
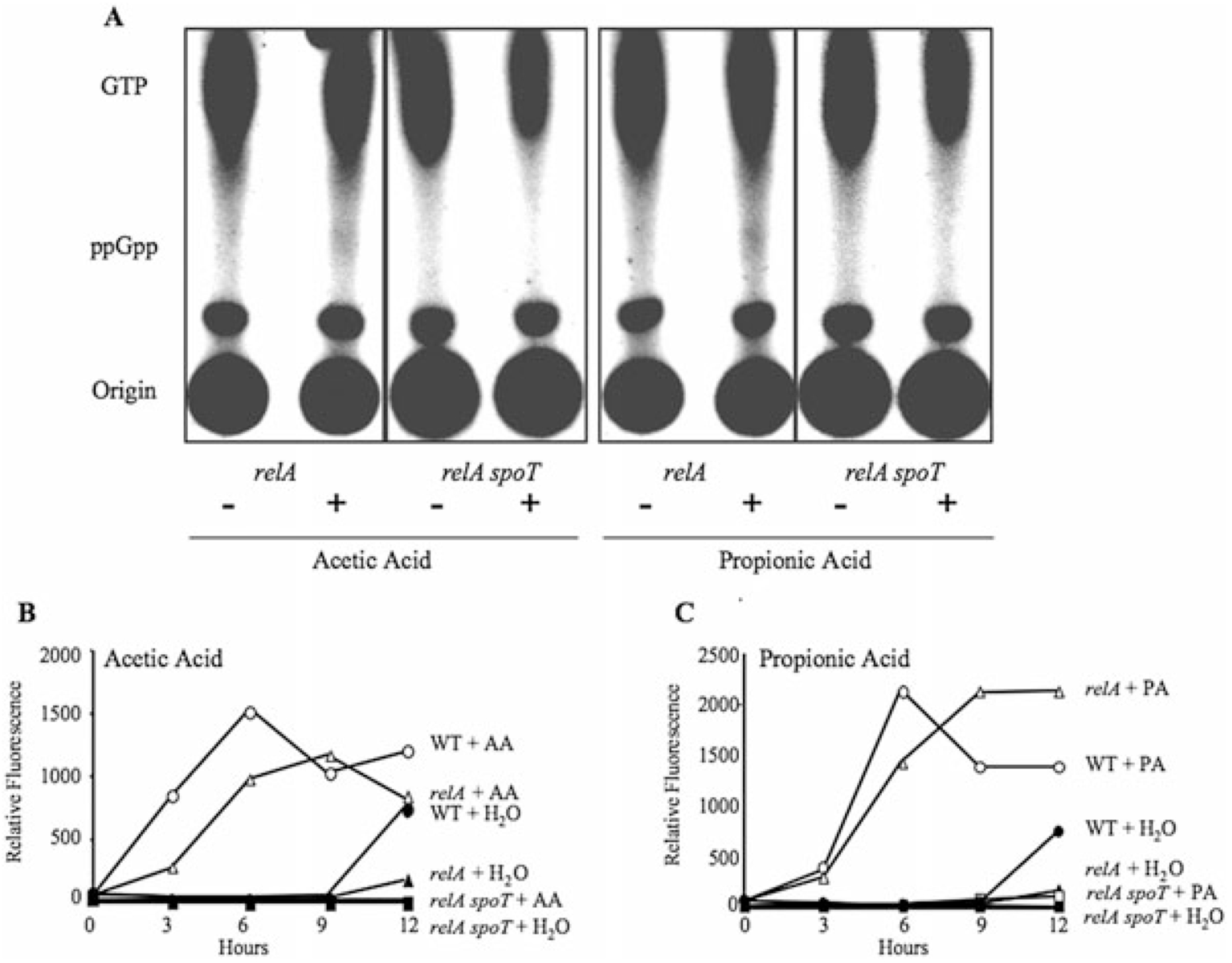
*L. pneumophila* employs the stringent response to induce differentiation when fatty acid biosynthesis is altered. A. Perturbations in fatty acid biosynthesis trigger ppGpp production in the E phase. After labelling nucleotide pools with ^32^P, E cultures of *relA* and *relA spoT* mutant *L. pneumophila* were supplemented with water or 10 mM SCFAs for 1.5 h to stimulate ppGpp synthesis. As ppGpp is synthesized from GTP and low levels of ppGpp are difficult to detect by TLC, the GTP pools are also indicated for each sample. Representative chromatograms from two or more independent experiments are shown for each condition. B and C. *L. pneumophila* requires SpoT to sense SCFAs. E phase cultures of WT (circles), *relA* (triangles) or *relA spoT* (squares) *L. pneumophila* containing the *flaAgfp* reporter were supplemented with water (closed symbols), 10 mM acetic acid (AA) (B; open symbols) or 10 mM propionic acid (PA) (C; open symbols), and their fluorescence analysed at the times indicated. Shown are representative graphs from at least three independent experiments in which the mean fold change in fluorescence at 6 h ± SEM when compared with H_2_O controls was: WT+AA=23.6 ± 5.9; *relA* + AA = 30.7 ± 10.2; *relA spoT* + AA = 3.4 ± 1.9; WT+ PA = 48.5 ± 16.3; *relA* + PA = 47.5 ± 16.0; *relA spoT* + PA = 4.7 ± 2.6.

Many microbes produce ppGpp from GTP to adapt to nutritional and metabolic stresses such as deprivation of amino acids, carbon, iron, phosphorous and fatty acids (Srivatsan and Wang, 2008). Moreover, a regulatory role for ACP in the stringent response has recently been described in *E. coli*: SpoT directly interacts with the functional form of ACP, and single amino acid substitutions that disrupt this interaction abrogates SpoT-dependent ppGpp accumulation when fatty acid biosynthesis is inhibited (Battesti and Bouveret, 2006). Likewise, when fatty acid biosynthesis is inhibited pharmacologically in *L. pneumophila*, ppGpp accumulates by a mechanism that requires SpoT, but not RelA (Dalebroux et al., 2009). Therefore, we investigated whether *L. pneumophila* also accumulates ppGpp in response to excess SCFAs. When supplemented with either 10 mM acetic or propionic acid, *relA* mutant cultures exhibited a trace level of ppGpp when compared with either the water or the *relA spoT* control (Fig. 7A; Table 1). The weak ppGpp signal detected in *relA* cells is consistent with that observed when WT and *relA* mutant *L. pneumophila* are treated with cerulenin, an inhibitor of fatty acid biosynthesis (Dalebroux et al., 2009; data not shown). Because the slight ppGpp accumulation by *L. pneumophila* exposed to SCFAs was not conclusive, we analysed genetically whether either the RelA or SpoT enzyme was required to co-ordinate this differentiation.

When E cultures were supplemented with either 10 mM acetic or propionic acid, *relA* mutant *L. pneumophila* differentiated, similar to WT (Fig. 7B and C). In contrast, *relA spoT* mutants were unable to trigger the phenotypic switch (Fig. 7B and C). Together, our phenotypic, biochemical and genetic data presented both here and elsewhere (Dalebroux et al., 2009) demonstrate that, when fatty acid biosynthesis is perturbed, SpoT equips *L. pneumophila* to invoke the stringent response pathway to initiate a swift differentiation programme and rapidly adapt to metabolic stress.

## Discussion

**Fig. 8.**
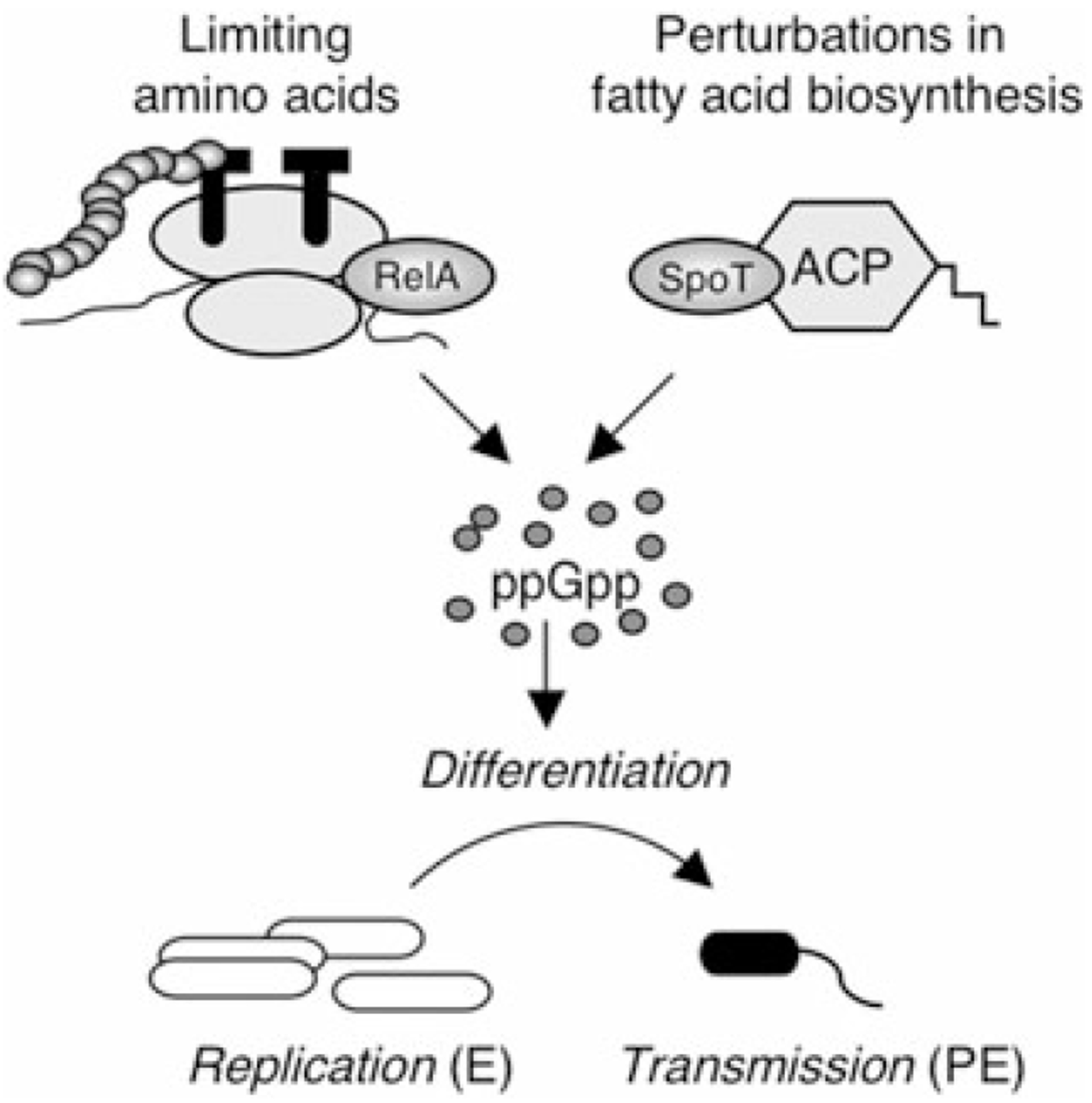
*L. pneumophila* monitors flux in fatty acid biosynthesis to co-ordinate differentiation. See text for discussion of the model.

Because *L. pneumophila* persist within many diverse environments, we predicted that various metabolites cue its differentiation. By screening several hundred compounds via Phenotype MicroArrays, we identified 22 inducers of *L. pneumophila* differentiation and focused on carboxylic acids, which trigger a premature transition from the replicative to the transmissive phase (Fig. 2, Tables 2 and 3 and data not shown). Previous studies postulated that when amino acid concentrations are limiting, uncharged tRNAs accumulate and the RelA enzyme synthesizes ppGpp, an alarmone that activates the regulatory cascade that governs *L. pneumophila* differentiation (Fig. 8; Hammer and Swanson, 1999; Zusman et al., 2002). We have expanded this model by showing that SpoT co-ordinates transmission trait expression either when fatty acids are excessive or when their biosynthesis is perturbed, likely mediated by a regulatory interaction between SpoT and ACP (Figs 7 and 8; Seyfzadeh et al., 1993; Battesti and Bouveret, 2006; Dalebroux et al., 2009). Genetic data suggest that when SpoT can no longer bind ACP, *L. pneumophila* fails to differentiate in response to alterations in fatty acid biosynthesis (Dalebroux et al., 2009). Thus, we extend the paradigm of microbial differentiation by reporting that the stringent response machinery equips *L. pneumophila* to monitor both protein and fatty acid biosynthesis to regulate its virulence expression and govern transmission.

The mechanism by which bacteria detect fluctuations in fatty acid biosynthesis remains to be elucidated. In *E. coli*, SpoT might sense either an accumulation or a depletion of an intermediate in this biosynthetic pathway (DiRusso and Nystrom, 1998; Battesti and Bouveret, 2006). For *Bacillus subtilis*, a key regulator of lipid metabolism is malonyl-CoA, a molecule that may act as a signal during stress and starvation (Schujman et al., 2008). Similarly, E phase *L. pneumophila* immediately induce the *flaAgfp* reporter when treated with cerulenin (Fig. 5D), an inhibitor of fatty acid biosynthesis that causes malonyl-CoA to accumulate (Heath and Rock, 1995). On the other hand, TOFA, which is predicted to deplete the levels of malonyl-CoA present in the cell (Cook et al., 1978; McCune and Harris, 1979), fails to stimulate E phase *L. pneumophila* to activate the *flaA* promoter (Fig. 5B, C and E). Therefore, *L. pneumophila* may monitor the levels of malonyl-CoA in the cell to regulate its phenotypic switch.

Alternatively, *L. pneumophila* may gauge the acyl chains attached to ACP. Perhaps the bacteria recognize either an accumulation or a depletion of one or more of the acyl-ACP species (e.g. Fig. 6) or an altered ratio of acyl-ACP to apo-ACP. In *E. coli*, there are numerous intermediates in the fatty acid biosynthetic pathway; accordingly, more detailed studies are needed to determine which, if any, intermediate(s) triggers *L. pneumophila* differentiation. *L. pneumophila* also encodes three putative ACPs (*lpg0359*, *lpg1396* and *lpg2233*) that are each predicted to be modified by 4′-phosphopantetheine (Magnuson et al., 1993). Our data do not address which ACP(s) is involved, as the specificity of the ACP antibody has not been determined (Fig. 6). Therefore, whether each ACP plays a unique or redundant role in the *L. pneumophila* life cycle remains to be determined.

By analogy to *E. coli*, we favour a model by which ppGpp-dependent sigma factor competition enables *L. pneumophila* to fine-tune its gene expression profile (reviewed by Bachman and Swanson, 2004a; Magnusson et al., 2005). The quantity of ppGpp observed in response to SCFAs and cerulenin is considerably less than that of PE bacteria (Fig. 7A; Dalebroux et al., 2009). This is consistent with the previous report that *E. coli* produces low levels of ppGpp in response to fatty acid starvation (Seyfzadeh et al., 1993). The difficulty in detecting ppGpp may reflect our labelling conditions: Due to its fastidious nature, a phosphate-limited medium to label nucleotides efficiently is not a viable option for *L. pneumophila* studies. Nevertheless, since every PE trait is induced when E phase *L. pneumophila* are treated with excess SCFAs or cerulenin, even a modest level of ppGpp may be sufficient to trigger differentiation (Fig. 2, Table 3). Presumably, when fatty acid biosynthesis is altered, *L. pneumophila* produces a quantity of ppGpp sufficient to recruit to RNA polymerase the appropriate cohort of its six alternative sigma factors (Cazalet et al., 2004; Chien et al., 2004) to induce the PE traits that promote transmission to a new host and survival in the environment.

Several circumstances could alter the quantity of fatty acids in *L. pneumophila*’s intracellular niche. When the TCA cycle does not operate completely, or when bacterial cells are flooded with excess carbon, microbes excrete acetate into their extracellular milieu (Wolfe, 2005). *L. pneumophila* also possesses lipolytic enzymes that may generate free fatty acids by degrading membranes of their own or their host (Hood et al., 1986; Archuleta et al., 2005). Alternatively, *L. pneumophila* may monitor external sources of fatty acids that are derived from the host plasma or phagosomal membranes. Consistent with this idea, within A/J mouse macrophages *L. pneumophila* replicate within a lysosomal compartment (Sturgill-Koszycki and Swanson, 2000), the site for membrane degradation. Interestingly, the alveolar macrophages of rats can ingest pulmonary surfactant, which is rich in phosphatidylcholine and phosphatidylglycerol (Grabner and Meerbach, 1991), two substrates for the phospholipase A secreted by *L. pneumophila* (Flieger et al., 2000). By this scenario, the accidental human host may exacerbate pathogenesis by stimulating synthesis of the transmission factor flagellin, which provokes a macrophage inflammatory cell death pathway (Molofsky et al., 2006; Ren et al., 2006; Vinzing et al., 2008).

Additional metabolites that are present on the Biolog arrays may also cue intracellular differentiation of *L. pneumophila*. First, the permeability of *L. pneumophila* for each of the compounds on the Phenotype MicroArrays is unknown. Also, the plates include one concentration of each compound, yet titration curves indicate that several inducers only trigger differentiation within a narrow concentration range (data not shown). Indeed, nicotinic acid, which is present on Biolog plate PM5 at 10 μM, did not cause growth restriction or induction of the *flaAgfp* reporter, whereas 5 mM nicotinic acid does trigger *L. pneumophila* differentiation, as judged by both micro-array and phenotypic data (R.L. Edwards, M. Jules, C. Buchrieser and M.S. Swanson, unpublished).

*Legionella pneumophila* can monitor perturbations in fatty acid biosynthesis to regulate its differentiation *in vitro*, but whether SCFAs also induce transmission traits within vacuoles of phagocytic cells is not known, as their composition has yet to be elucidated. Although *relA* is dispensable for intracellular growth in both human macrophages and amoebae (Hammer and Swanson, 1999; Zusman et al., 2002), *L. pneumophila* does require SpoT not only for transmission between mouse macrophages, but also to differentiate from the replicative to the transmissive phase (Dalebroux et al., 2009). Therefore, *L. pneumophila* employs SpoT to monitor fatty acids or some other metabolite in macrophage vacuoles to govern its life cycle. By linking central metabolism to differentiation and virulence, *L. pneumophila* augments its fitness by adapting to fluctuating environments.

## Experimental procedures

### Bacterial strains and cultures

*Legionella pneumophila* strain Lp02 (*thyA hsdR rpsL*; MB110), a virulent thymine auxotroph, was the parent for all strains constructed (Berger and Isberg, 1993; Table 1). To obtain *letA* and *letS* mutants lacking pflaG (MB413 and MB416 respectively), the mutant alleles from MB414 and MB417 were transferred onto the Lp02 chromosome by natural competence (Hammer et al., 2002). *relA* (MB696) and *relA spoT* (MB697) mutants were generated using standard techniques as described elsewhere (Dalebroux et al., 2009). To monitor the induction of the *flaA* promoter, the *relA* and *relA spoT* mutants were transformed with the pflaG plasmid to generate MB684 and MB685 respectively.

To construct a *pta ackA2* (*lpg2261* and *lpg2262*) deletion mutant, the 3.3 kb *pta ackA2* locus was amplified from Lp02 genomic DNA using forward primer 5′-GCAACTCGTATGCCATAC and reverse primer 5′-GTAAATCCATCGCTTTGGG. The PCR fragment was purified and ligated to pGEM-T (Promega), transformed into *E. coli* DH5a, and the resulting plasmid designated as pGEM-T-PtaAckA2 (MB619). A 1.8 kb region of the *pta ackA2* open reading frame was removed by digestion with XmaI and NheI, and the remaining pGEM-T-PtaAckA2 fragment was blunted with Klenow and treated with Antarctic phosphatase (New England Biolabs). The 1.3 kb kanamycin-resistance cassette from pUC4K was removed via EcoRI digestion, blunted with Klenow and ligated into the digested pGEM-T-PtaAckA2 plasmid to create pGEM-T-PtaAckA2::Kan (MB681). After verification by PCR, the deletion/insertion alleles were transformed into Lp02 via natural competence and selected for by antibiotic resistance (Stone and Abu Kwaik, 1999). The desired chromosomal mutation was confirmed by PCR and the resulting strain designated as MB641. To monitor the induction of the *flaA* promoter by fluorometry, MB641 was transformed with pflaG. Two independent isolates were tested in fluorometry assays and found to be similar; MB682 data are displayed.

Bacteria were cultured at 37°C in 5 ml aliquots of *N-*(2-acetamido)-2-aminoethanesulphonic acid (ACES; Sigma)-buffered yeast extract (AYE) broth and supplemented with 100 μg ml^−1^ thymidine when necessary. Cultures having an optical density at 600 nm (OD_600_) of 0.5–0.85 were defined as exponential (E), and those of OD_600_ 3.4–4.5 as post-exponential (PE). To obtain cfu, *L. pneumophila* were plated on ACES-buffered charcoal-yeast extract agar supplemented with 100 μg ml^−1^ thymidine (CYET) and incubated at 37°C for 4–5 days.

### Biolog Phenotype MicroArray analysis

Phenotype MicroArray (PM) plates were purchased from Biolog (Hayward, CA; Table 2). One hundred microlitres of E phase MB355 cultured in AYE media was added to each well of the Biolog plates, and the plates were incubated at 37°C while shaking. After 3 or 6 h, 100 μl of each culture was transferred to black, clear-bottom tissue culture plates (Costar), and the relative fluorescence intensity was quantified using a Synergy HT microplate reader (Bio-Tek) using 485 nm excitation, 530 nm emission and sensitivity of 50. Inducers were defined as having a 1.4–7.3 mean fold increase in fluorescence at 6 h when compared with the negative control well of the Biolog plates in at least three independent experiments.

### Fluorometry

To monitor expression of the flagellin promoter, *L. pneumophila* strains containing the *flaAgfp* reporter plasmid pflaG were cultured in AYE media. At OD_600_ = 0.50–0.85 (T = 0), the cultures were supplemented with 10 mM acid, 0.5 μg ml^−1^ cerulenin (Sigma) or 5 μg ml^−1^ TOFA (Cayman Chemical). Cultures supplemented with water or DMSO served as negative and vehicle controls respectively. At the times indicated, culture cell density was measured as OD_600_. To analyse similar bacterial numbers, aliquots were collected by centrifugation, and the cell densities were normalized to OD_600_ = 0.01 in PBS. The fluorescence intensity of a 200 μl aliquot was measured as described above.

### Motility

To qualitatively assess motility, 10 μl wet mounts of broth-grown *L. pneumophila* were prepared and immediately examined by phase-contrast microscopy. Relative motility levels were based on at least three independent observations of fields that contained several hundred microbes. A score of (−) was assigned to cultures that were <10% motile; (+) indicates cultures that were 10–25% motile; (++) indicates cultures that were 25–75% motile; and (+++) indicates that > 75% of the microbes within the fields displayed high levels of directed motility.

### Macrophages

Macrophages isolated from femurs of female A/J mice (Jackson Laboratory) were cultured in RPMI-1640 containing 10% heat-inactivated fetal bovine serum (RPMI/FBS; Gibco BRL) as described previously (Swanson and Isberg, 1995). Following a 7 day incubation in medium supplemented with l-cell supernatant, macrophages were plated at either 5 × 10^4^ or 2.5 × 10^5^ per well for cytotoxicity and degradation assays respectively.

### Cytotoxicity

To measure contact-dependent cytotoxicity of *L. pneumophila* for macrophages, PE bacteria or E phase cultures supplemented with water or 10 mM fatty acids for 3 h were added to monolayers at the indicated multiplicities of infection (moi). After centrifugation at 400 *g*for 10 min at 4°C (Molofsky et al., 2005), the cells were incubated for 1 h at 37°C. To quantify macrophage viability, RPMI/FBS containing 10% alamarBlue (Trek Diagnostic Systems) was added to the monolayers for 6–12 h, and the reduction of the colorimetric dye was measured spectrophotometrically as described (Byrne and Swanson, 1998; Hammer and Swanson, 1999; Molofsky et al., 2005).

### Degradation

The percentage of intracellular *L. pneumophila* that remained intact after a 2 h macrophage infection was quantified by fluorescence microscopy. Briefly, macrophages were plated at 2.5 × 10^5^ onto coverslips in 24 well plates. Then, PE bacteria or E phase microbes exposed to either water or 10 mM fatty acids for 3 h were added to macrophage monolayers at a moi of μ1. The cells were centrifuged at 400 *g* for 10 min at 4°C and then incubated for 2 h at 37°C. Uninternalized bacteria were removed by washing the monolayers with 3 × 0.5 ml RPMI/FBS. Control experiments indicate that <1% of the cell-associated bacteria are extracellular following the three RPMI/FBS washes (Bachman and Swanson, 2004a; and data not shown). Macrophages were then fixed, permeabilized and stained for *L. pneumophila* as described (Bachman and Swanson, 2001).

### Sodium sensitivity

To calculate the percentage of *L. pneumophila* that are sensitive to sodium, PE bacteria or E cultures supplemented with either water or 10 mM fatty acids for 3 h were plated onto CYET with or without 100 mM NaCl. After 6 days at 37°C, cfu were enumerated and the percentage of sodium sensitive microbes calculated as described (Byrne and Swanson, 1998).

### Analysis of acyl-ACPs

For purification of acyl-ACPs, WT *L. pneumophila* were cultured to the E phase at 37°C on an orbital shaker in 250 ml AYE containing 100 μg ml^−1^ thymidine. Upon reaching an OD_600_ between 0.5 and 0.85, the cultures were supplemented with water, 10 mM fatty acid or 0.5 μg ml^−1^ cerulenin and then cultured for an additional 3 h. After collection by centrifugation at 4000 *g* for 20 min at 4°C, the cell pellets were stored at −80°C. Once thawed on ice, the pellets were resuspended in 12.5 ml ACP buffer (200 mM NaCl, 20 mM Tris-HCl, pH 6, 1 mM EDTA). To reduce protein degradation, one tablet of a protease inhibitor cocktail (Roche) was added to each 12.5 ml suspension. Cells were lysed by sonication and the lysates cleared by centrifugation at 7000 *g* for 1 h at 4°C. As *L. pneumophila* is predicted to contain three ACPs ranging from 8.6 to 15.3 kDa, large-molecular-weight proteins were removed from the lysates via 50K and 30K centrifugal filter devices (Amicon Ultra, Millipore # UFC905024 and UFC903024 respectively). The remaining ACP fractions were concentrated with 5K centrifugal filter devices (# UFC800524), which also removed small-molecular-weight proteins and salts. The protein concentration of each sample was determined using the Bio-Rad Protein Assay, and samples were stored at −20°C. To visualize the profile of intracellular acyl-ACPs, 13% non-denaturing gels were prepared, and urea was added to either 0.5 M or 2.5 M for SCFA or long-chain fatty acid gels respectively (Rock and Cronan, 1981; Jackowski and Rock, 1983; Post-Beittenmiller et al., 1991). After electrophoresis in 192 mM glycine, 25 mM Tris buffer, samples were transferred to polyvinylidene difluoride membrane (Bio-Rad), and the membranes blocked in TBS-T (20 mM Tris-HCl, pH 7.5, 500 mM NaCl, 0.05% Tween 20) containing 5% non-fat milk. To detect the *L. pneumophila* ACP proteins, the membranes were probed with a primary antibody generated by *E. coli* ACP (gift from C.O. Rock, Memphis, TN) diluted 1:500 and a secondary goat anti-rabbit antibody conjugated to horseradish peroxidase (Pierce) diluted 1:8000 (Jackowski and Rock, 1983) and then developed with SuperSignal West Pico Chemiluminescent Substrate (Pierce).

### Detection of ppGpp

Accumulation of the ppGpp signalling molecule in response to flux in fatty acid metabolism was detected by thin-layer chromatography (TLC) as described (Cashel, 1969; 1994; Hammer and Swanson, 1999). Briefly, E phase *relA* (MB696) and *relA spoT* (MB697) cultures were diluted to an OD_600_ = 0.25 and cultured at 37°C on a roller drum with approximately 100 μCi ml^−1^ carrier-free [^32^P]-phosphoric acid (ICN Pharmaceuticals) for 6 h, or two generation times. Next, cultures were supplemented with water, 10 mM acetic acid or 10 mM propionic acid and incubated for an additional 1.5 h at 37°C. To extract the nucleotides, 50 μl aliquots were removed from each culture and added to 13 M formic acid and then incubated on ice for 15 min. Samples were subjected to two freeze-thaw cycles and stored at −80°C until used for chromatography. Formic acid extracts (25 μl) were applied to a PEI-cellulose TLC plate (20 × 20) and developed with 1.5 M KH_2_PO_4_, pH 3.4 as described (Cashel, 1969; 1994; Hammer and Swanson, 1999). TLC plates were exposed to autoradiography film for 72 h and developed in a phosphoimager. To monitor growth following water or fatty acid supplementation, optical densities were determined for non-radioactive cultures grown under identical conditions.

## Figures and Tables

**Table 1.** Bacterial strains and plasmids.

Strain or plasmid	Relevant genotype/phenotype	Reference or source
Strains		
*E. coli*		
DH5α	F^−^ *endA1 hsdR17* (r^−^ m^+^) *supE44 thi-l recA1 gryA* (Nal^1^) *relA1*Δ (l*acZYA-argF*^−^)*_U169_* ϕ80d*/acZ*Δ*M15* λ*pir*RK6	Laboratory collection
MB619	DH5α pGEM-T-PtaAckA2	This work
MB681	DH5α pGEM-T-PtaAckA2::Kan	This work
*L. pneumophila*		
MB110	Lp02 wild-type; *thyA hsdR rpsL*	Berger and Isberg (1993)
MB355	pflaG	Hammer and Swanson (1999)
MB413	*letA-22::kan*	Hammer et al. (2002)
MB414	*letA-22::kan* pflaG	Hammer et al. (2002)
MB416	*letS-36::kan*	Hammer et al. (2002)
MB417	*letS-36::kan* pflaG	Hammer et al. (2002)
MB641	*pta ackA2*::*kan*	This work
MB682	*pta ackA2*::*kan* pflaG	This work
MB684	*relA*::*kan* pflaG	Dalebroux et al. (2009)
MB685	*relA*::*gent spoT*::*kan* pflaG	Dalebroux et al. (2009)
MB696	*relA*::*kan*	Dalebroux et al. (2009)
MB697	*relA*::*gent spoT*::*kan*	Dalebroux et al. (2009)
Plasmids		
pGEM-T	Multiple cloning site within coding region of β-lactamase α fragment linearized with single-T overhangs; 3 kb; Amp^R^	Promega
pflaG	150 bp *flaA* promoter fragment fused to green fluorescent protein encodes thymidylate synthetase; 10.5 kb; Amp^R^	Hammer and Swanson (1999)
pGEM-T-PtaAckA2	pGEM-T containing 3.3 kb *pta ackA2* locus PCR amplified from Lp02 chromosome and ligated into T overhangs; 6.3 kb; Amp^R^	This work
pUC4K	pUC4 containing 1.3 kb kanamycin cassette	Pharmacia
pGEM-T-PtaAckA2::Kan	pGEM-T-PtaAckA2 with 1.3 kb kanamycin cassette inserted between XmaI and NheI sites in the *pta ackA2* open reading frame resulting in a 1.8 kb deletion	This work

**Table 2.** Compounds that trigger premature differentiation in *L. pneumophila*.

Compound^a^	Biolog plate	Mean FC^b^ at 6 h
Carboxylic acids		
Formic acid	PM1	7.3 ± 0.6
Acetic acid	PM1	1.8 ± 0.2
Propionic acid	PM1	4.8 ± 1.9
Butyric acid	PM2A	3.3 ± 1.9
α-Ketovaleric acid	PM2A	1.9 ± 0.7
Caproic acid	PM2A	7.2 ± 1.4
Itaconic acid	PM2A	2.4 ± 0.8
Sorbic acid	PM2A	2.6 ± 0.4
4-hydroxybenzoic acid	PM2A	1.8 ± 0.4
m-hydroxy phenyl acetic acid	PM1	3.8 ± 2.1
p-hydroxy phenyl acetic acid	PM1	2.9 ± 1.4
Monomethyl succinate	PM1	4.7 ± 0.6
Detergents		
Polyoxyethylene sorbitan monolaurate (Tween 20)	PM5	3.2 ± 1.2
Polyoxyethylene sorbitan monooleate (Tween 80)	PM1	1.4 ± 0.1
Other		
2-Deoxy-d-glucose 6-phosphate	PM4A	2.0 ± 1.3
Deoxyadenosine	PM1	2.0 ± 0.2
Deoxyribose	PM2A	2.3 ± 0.4
Dihydroxyacetone	PM2A	2.7 ± 0.4
Hydroxylamine	PM3B	1.7 ± 0.2
Met-Ala dipeptide	PM3B	1.6 ± 0.4
Nitrite	PM3B	5.1 ± 1.8
Parabanic acid	PM3B	1.5 ± 0.3

a. Approximate concentrations: 5–20 mM carbon sources, 2–5 mM nitrogen sources, and 0.1–2 mM phosphorus and sulphur sources.

b. FC indicates the fold change ± SD in fluorescence between the compound and the negative control well on the Phenotype MicroArray plate.

**Table 3.** Phenotypic response of *letA* and *letS* mutants 3 h after fatty acid supplementation.

Strain	Culture conditions	Growth inhibition^a^	Motility^b^	Cytotoxicity^c^	Degradation^d^	Na sensitivity^e^
WT	PE Control	+	+++	+ (9 ± 2%)	+ (82 ± 2%)	+ (0.3 ± 0.2%)
	E + H_2_O	−	−	−(68 ± 15%)	−(16 ± 2%)	−(85 ± 13%)
	E + acetic acid	+	++	+(16 ± 3%)	+(52 ± 5%)	+(8 ± 2%)
	E + propionic acid	+	++	+(23 ± 8%)	+(62 ± 5%)	+(9 ± 3%)
*letA*	PE control	+	−	−(74 ± 13%)	−(38 ± 5%)	−(48 ± 21%)
	E + H_2_O	−	−	−(78 ± 12%)	−(18 ± 3%)	−(118 ± 16%)
	E + acetic acid	+	−	−(65 ± 2%)	−(27 ± 3%)	−(14 ± 7%)
	E + propionic acid	+	−	−(76 ± 9%)	−(35 ± 7%)	−(18 ± 7%)
*letS*	PE control	+	−	−(82 ± 10%)	−(47 ± 5%)	−(64 ± 6%)
	E + H_2_O	−	−	−(81 ± 8%)	−(18 ± 3%)	−(105 ± 4%)
	E + acetic acid	+	−	−(71 ± 15%)	−(29 ± 2%)	−(22 ± 10%)
	E + propionic acid	+	−	−(69 ± 16%)	−(41 ± 3%)	−(26 ± 20%)

a. Growth of *L. pneumophila* was monitored by measuring the OD_600_ of the cultures 3 h after supplementation. Although *letA* and *letS* cultures supplemented with fatty acids do not display PE phenotypes, bacterial growth is completely inhibited. (+) indicates growth inhibition, while (−) indicates normal growth kinetics. Data represent at least three independent experiments.

b. Motility was assessed by phase-contrast microscopy and is based on numerous independent observations. (−) indicates cultures that were < 10% motile (+) indicates 10–25% motility (++) indicates 25–75% motility, and (+++) indicates high levels of directed motility (> 75%).

c. Cytotoxicity of *L. pneumophila* for macrophages was assessed by measuring the reduction of alamarBlue following a 1 h incubation. Data points that fell within a moi range of 5–10 were pooled. (+) indicates that less than 50% of the macrophages were viable, whereas (−) represents greater than 50% macrophage viability. In parentheses, the mean percent of viable macrophages ± SEM is shown from at least three independent experiments performed in triplicate.

d. The per cent of bacteria that remain intact following a 2 h incubation within macrophages was determined by fluorescence microscopy. (+) indicates that > 50% of the bacteria were able to avoid degradation and (−) indicates that < 50% of the bacteria avoided degradation. Data represent the mean ± SEM from at least three independent experiments performed in duplicate.

e. The per cent of sodium sensitive bacteria was calculated by comparing cfu of cultures plated onto media with and without 100 mM NaCl. (+) indicates < 10% inhibition and (−) indicates > 10% inhibition. The values represent the mean ± SEM for at least three independent experiments performed in duplicate.
